# Fatal Hyperphosphatemia After a Single Phosphate Enema in Ogilvie Syndrome

**DOI:** 10.7759/cureus.101583

**Published:** 2026-01-15

**Authors:** Isabel Monteiro, Susana Viana, Natália Pires, Liliana Carneiro

**Affiliations:** 1 Internal Medicine Service, Pedro Hispano Hospital, Matosinhos Local Health Unit, Matosinhos, PRT

**Keywords:** acute colonic pseudo-obstruction, electrolyte disturbances, hyperphosphatemia, ogilvie syndrome, phosphate enema

## Abstract

Phosphate-containing enemas are commonly used for constipation and fecal impaction but may cause severe electrolyte disturbances when retained, particularly in patients with impaired intestinal motility. We report a fatal case of extreme hyperphosphatemia after a single phosphate enema in a 75-year-old woman with acute colonic pseudo-obstruction (Ogilvie syndrome). Despite normal baseline renal function, the patient rapidly developed profound metabolic derangements, including marked hyperphosphatemia, severe hypocalcemia, hypernatremia, hyperkalemia, and metabolic acidosis, leading to distributive shock and multiorgan failure. This case highlights the heightened risk of phosphate absorption in pseudo-obstruction, where colonic dilation and potential mucosal ischemia may markedly increase epithelial permeability. Clinicians should avoid phosphate-containing enemas in elderly or high-risk patients with impaired intestinal motility and prioritize safer decompression strategies.

## Introduction

Phosphate-containing enemas are widely used for constipation and fecal impaction, particularly in elderly or immobilized patients. Although generally safe when promptly expelled, they may cause severe electrolyte disturbances, such as hyperphosphatemia, hypocalcemia, hypernatremia, and acute kidney injury, when retained in the colon [1-3]. Impaired intestinal motility is a major risk factor, as prolonged intraluminal contact substantially increases phosphate absorption. Conditions associated with colonic dilation and mucosal injury, such as acute colonic pseudo-obstruction and ischemic or inflammatory colitis, may further increase epithelial permeability and potentiate systemic phosphate uptake.

Acute colonic pseudo-obstruction (Ogilvie syndrome) is characterized by marked colonic dilation in the absence of mechanical obstruction and typically occurs in debilitated or hospitalized patients. Colonic distension, mucosal inflammation, and early ischemia in this setting may markedly enhance phosphate absorption, placing patients at particularly high risk when phosphate-containing enemas are administered [4]. Despite multiple reports documenting the risks associated with phosphate enemas, fatal outcomes following a single rectal dose have been described only in case reports and small single-center case series [5,6]. Clinician awareness of this risk is therefore limited. We report a fatal case of extreme hyperphosphatemia after a single phosphate enema in a patient with Ogilvie syndrome, highlighting the pathophysiological mechanisms and reinforcing the need for safer decompression strategies in high-risk patients [7].

## Case presentation

A 75-year-old woman, previously autonomous and not taking any chronic medication, including laxatives, presented to the emergency department with a one-month history of worsening constipation, progressive abdominal distension, and nonspecific abdominal discomfort. Her medical history was notable for chronic constipation and a prior hospitalization for intestinal obstruction, which resolved with conservative medical management without the need for surgical intervention. She denied fever but reported nausea and a single episode of dark-colored emesis.

On admission, she appeared dehydrated, with dry oral mucosa and marked cachexia, but was afebrile, with a blood pressure of 143/85 mmHg and a heart rate of 79 beats per minute (bpm). The abdomen was markedly distended and tympanic, with moderate diffuse tenderness on palpation, without guarding or peritoneal signs. Bowel sounds were present and metallic in character. Digital rectal examination revealed a large fecaloma.

Initial laboratory evaluation demonstrated preserved renal and hepatic function, with normal transaminase levels and serum electrolytes, including calcium, phosphate, sodium, and potassium (Table 1). Arterial blood gas analysis was unremarkable. Given the marked abdominal distension, fecal impaction, and elevated inflammatory markers, empirical antimicrobial therapy was initiated due to concern for a possible early intra-abdominal infectious process.

**Table 1:** Laboratory parameters at admission and after clinical deterioration.

	Normal reference range	Admission	After 36 hours
Hemoglobin (g/dL)	12.0-16.0	13.7	11.1
White blood cell count (×10^3^/µL)	4.00-11.00	20.6	11.59
Neutrophils (×10^3^/µL)	1.3-8.8	19.1	10.8
Platelet count (×10^3^/µL)	150-400	436	311
Creatinine (mg/dL)	0.6-1.1	1.2	2.5
Urea (mg/dL)	21-43	50	95
Potassium (mEq/L)	3.4-5.1	4.4	6
Sodium (mEq/L)	136-145	143	156
Phosphorus (mg/dL)	2.3-4.7	3.9	33.3
C-reactive protein (mg/dL)	<5.00	116.6	311.7
pH	7.38-7.45	7.39	7.31
pCO_2_ (mmHg)	35.0-45.0	36	38
pO_2_ (mmHg)	95.0-100.0	86	77
HCO_3_^- ^(mmol/L)	22-26	22.8	19.1
Lactate (mmol/L)	0.5-2.2	1.2	1.6
Calcium (mmol/L)	1.12-1.32	1.18	0.35
Chloride (mmol/L)	98-107	107	103
Anion gap	10-14	13.2	32.8

**Figure 1:**
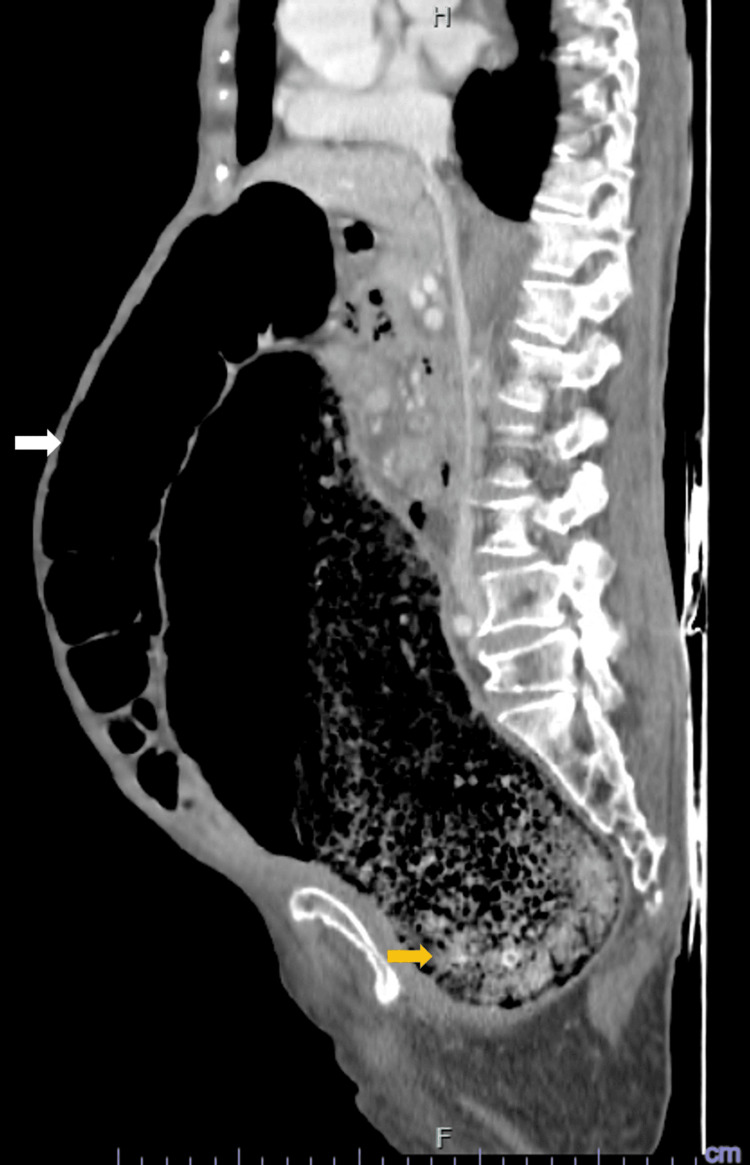
Sagittal CT findings in acute colonic pseudo-obstruction. Sagittal computed tomography (CT) image demonstrating marked colonic dilation (white arrow) with a large rectal fecaloma (yellow arrow), consistent with acute colonic pseudo-obstruction (Ogilvie syndrome).

**Figure 2:**
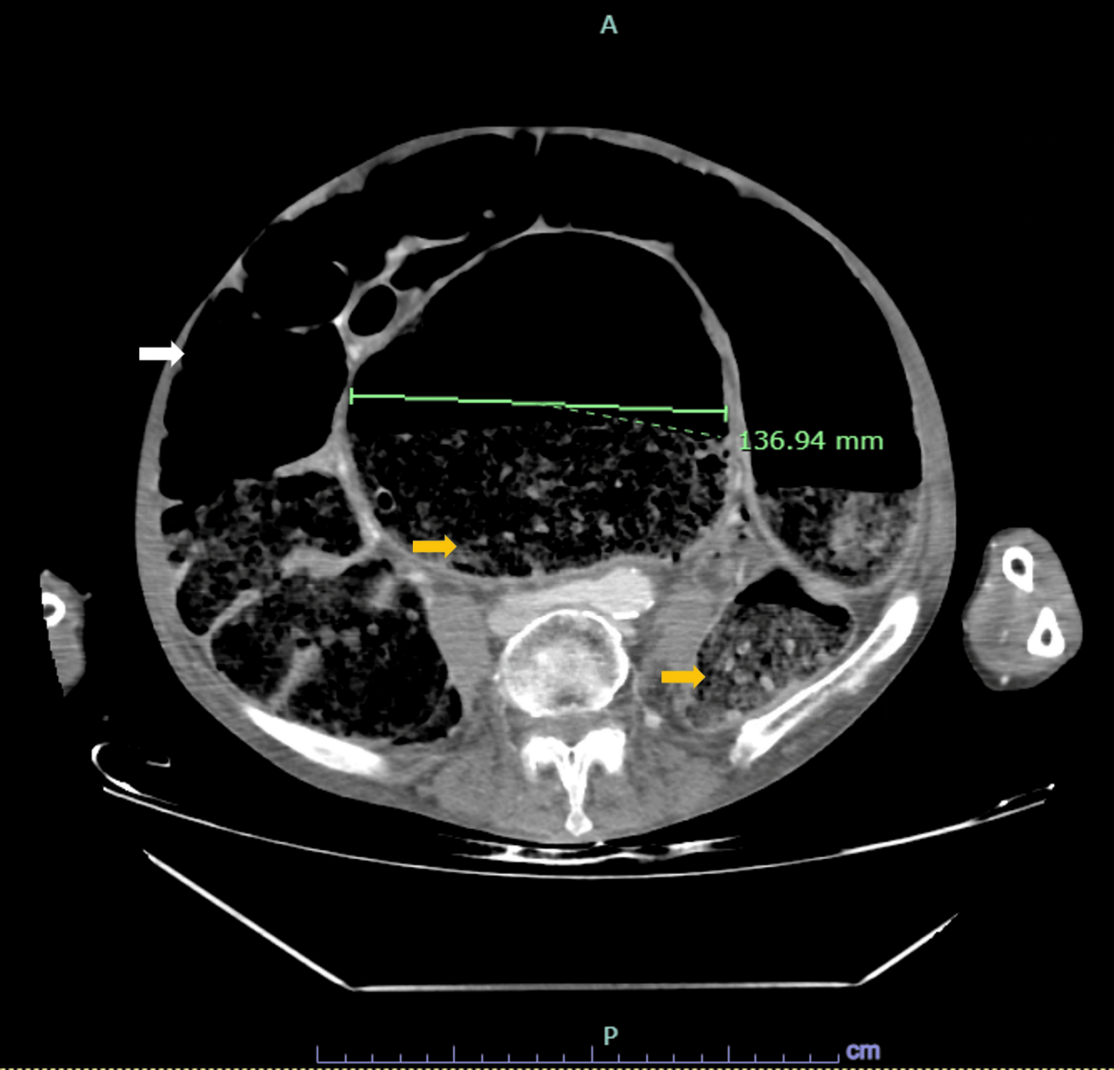
Axial CT findings in acute colonic pseudo-obstruction. Axial computed tomography (CT) image demonstrating marked colonic dilation (white arrow), with a measured transverse diameter of approximately 13.7 cm, and extensive fecal impaction consistent with a large rectal fecaloma (yellow arrows), in keeping with acute colonic pseudo-obstruction (Ogilvie syndrome).

Contrast-enhanced abdominal computed tomography revealed pronounced colonic dilation, with a sigmoid diameter of up to 13.6 cm, a large rectal fecaloma, and no evidence of mechanical obstruction, consistent with acute colonic pseudo-obstruction (Ogilvie syndrome) (Figures 1, 2).

Conservative management was initiated, including intravenous hydration and oral and rectal laxatives. Before the risk of phosphate retention in the setting of pseudo-obstruction was recognized, the patient received a commercially available sodium phosphate enema. Following enema administration, the patient was closely monitored with serial clinical assessments and laboratory testing. Partial stool evacuation occurred; however, significant colonic distension and constipation persisted, suggesting prolonged intraluminal retention.

Approximately 24 hours after enema administration, the patient developed progressive tachypnea, worsening abdominal distension, oliguria, and hemodynamic instability. Repeat laboratory testing revealed abrupt and severe metabolic derangements, including extreme hyperphosphatemia, profound hypocalcemia, worsening hypernatremia, hyperkalemia, and a high anion gap metabolic acidosis (Table 1).

Given the rapid clinical deterioration, the presence of advanced multiorgan dysfunction, and the evidence of severe frailty, both surgical and endoscopic decompression strategies were deemed unfeasible following multidisciplinary discussion. Despite aggressive fluid resuscitation, electrolyte correction, and supportive care, the patient progressed to distributive shock and multiorgan failure and died within 48 hours of hospital admission.

## Discussion

Acute colonic pseudo-obstruction (Ogilvie syndrome) creates a physiological setting that increases the risk of mucosal injury and prolonged luminal exposure, thereby facilitating systemic toxicity from phosphate-containing enemas. Under normal conditions, colonic phosphate absorption is limited, and excess phosphate is efficiently excreted by the kidneys. In Ogilvie syndrome, however, profound colonic hypomotility leads to the prolonged intraluminal retention of enema solutions, substantially increasing phosphate absorption. In addition, colonic distension, mucosal inflammation, and early ischemic changes may increase epithelial permeability, further facilitating rapid systemic phosphate uptake [4,7].

Once absorbed, excessive serum phosphate rapidly binds ionized calcium, leading to the formation of calcium-phosphate complexes and resulting in profound hypocalcemia. These complexes may deposit in vascular, muscular, and renal tissues, contributing to neuromuscular dysfunction, arrhythmias, hypotension, and acute kidney injury. Declining renal function further impairs phosphate excretion, creating a self-perpetuating cycle of worsening hyperphosphatemia, as described in previous reports of phosphate enema toxicity [1,3]. This cascade explains the abrupt metabolic collapse observed in our patient, who developed extreme hyperphosphatemia (33.3 mg/dL), severe hypocalcemia, hypernatremia, hyperkalemia, and high anion gap metabolic acidosis approximately 24 hours after enema administration.

Beyond massive phosphate absorption, additional factors likely amplified systemic deterioration in this case. Severe colonic distension may impair mucosal perfusion, leading to subclinical ischemia and further increasing epithelial permeability [4]. Moreover, the marked inflammatory response observed may have contributed to hemodynamic instability and transient impairment of renal phosphate clearance, exacerbating electrolyte derangements [2]. Partial stool evacuation following enema administration was insufficient to reduce intraluminal phosphate exposure, highlighting the unpredictable nature of phosphate absorption in the setting of impaired intestinal motility [5].

Several predisposing factors for phosphate enema toxicity described in the literature were present in this patient, including age of over 65 years, dehydration, chronic constipation, and the radiological evidence of severe colonic dilation [2,4]. Although baseline renal function was normal, prolonged intraluminal retention and mucosal compromise likely enabled massive phosphate absorption. Similar cases reported in the literature demonstrate that even a single phosphate enema can result in life-threatening toxicity when intestinal motility is impaired, particularly in elderly or hospitalized patients [5,6].

While severe toxicity is more frequently described after oral sodium phosphate preparations, rectal phosphate enemas are often perceived as safer. This case adds to the growing evidence that rectal phosphate administration can also precipitate catastrophic outcomes in high-risk patients [2,6]. Given the availability of safer alternatives, such as saline or oil-retention enemas, manual disimpaction, neostigmine, or endoscopic decompression, phosphate-containing enemas should be avoided in patients with suspected ileus or acute colonic pseudo-obstruction [7]. In severe or refractory cases, sigmoidoscopic decompression or surgical intervention may be required.

## Conclusions

This case demonstrates that even a single phosphate enema can precipitate severe electrolyte derangements and rapid clinical deterioration in patients with impaired intestinal motility. Acute colonic pseudo-obstruction markedly increases phosphate absorption, leading to life-threatening complications even in the absence of preexisting renal dysfunction. Phosphate-containing enemas should be avoided in high-risk patients, and safer decompression strategies should be prioritized to prevent similar fatal outcomes.
